# The investigation of frequency response for the magnetic nanoparticulate assembly induced by time-varied magnetic field

**DOI:** 10.1186/1556-276X-6-453

**Published:** 2011-07-14

**Authors:** Jianfei Sun, Yunxia Su, Chunyu Wang, Ning Gu

**Affiliations:** 1 State Key Laboratory of Bioelectronics, Southeast University, Nanjing 210096, PR China; 2 Jiangsu Key Laboratory of Biomaterials and Devices, Southeast University, Nanjing 210096, PR China; 3 Center of Materials Analysis, Nanjing University, Nanjing, 210093, PR China

**Keywords:** magnetic field, dynamic assembly, pattern formation, magnetic nanoparticles

## Abstract

The field-induced assembly of γ-Fe_2_O_3 _nanoparticles under alternating magnetic field of different frequency was investigated. It was found that the assembly was dependent upon the difference between colloidal relaxation time and field period. The same experiments on DMSA-coated γ-Fe_2_O_3 _nanoparticles exhibited that the relaxation time may be mainly determined by the magnetic size rather than the physical size. Our results may be valuable for the knowledge of dynamic assembly of colloidal particles.

## Background

With the expanding application of magnetic nanoparticles in cellular culture-matrix and tissue engineering, the interaction between nanomaterials and cells is becoming a central issue [1,2]. The assembly of magnetic nanoparticles will play an important role in the issue because the colloidal behavior can be greatly affected by the assembled morphology. Very recently, the time-varied (alternating) magnetic field got reported to be capable of inducing the assembly of iron oxide nanoparticles. It was discovered that Fe_3_O_4 _nanoparticles can form the fibrous assemblies in the presence of 80-KHz or 50-Hz alternating magnetic field [3,4]. The results also showed that the mechanism of colloidal assembly induced by the alternating magnetic field is essentially different from that induced by the static magnetic field, which may result from the variety in time domain. Thus, the frequency response of colloidal assembly directed by time-varied magnetic field is imperative to study. However, there has been little report on this topic.

In this paper, the experimental results of γ-Fe_2_O_3 _nanoparticulate assembly induced by alternating magnetic field of different frequency were presented. In the colloidal assembly induced by alternating magnetic field, the attractive force may arise from the interaction between two anti-parallel magnetic moments because the field is perpendicular to the assembly plane. Here, the strength of magnetic interaction is dependent upon the angle between two moment vectors. Now that the magnetic moments vary with external field during the assembly process, the frequency of external field may directly affect the magnetic interaction. Moreover, the nanoparticles often aggregate into clusters in aqueous suspension so that the state of magnetic coupling between nanoparticles is also vital for the magnetic interaction. In our experiments, two types of nanoparticles are employed to demonstrate the influence of magnetic coupling between nanoparticles on the field-directed assembly: bare γ-Fe_2_O_3 _nanoparticles and DMSA (meso-2,3-dimercaptosuccinic acid, HOOC-CH(SH)-CH(SH)-COOH)-coated γ-Fe_2_O_3 _nanoparticles.

## Results and discussion

**Figure 1:**
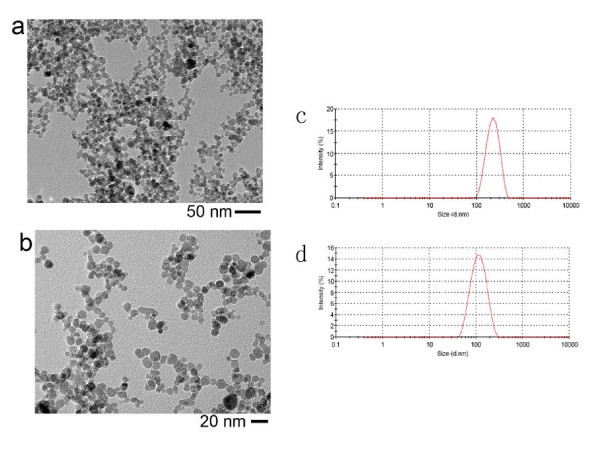
**TEM images of bare γ-Fe_2_O_3 _nanoparticles (a) and DMSA-coated nanoparticles (b)**. Dynamic light scattering measurements of bare γ-Fe_2_O_3 _nanoparticles (**c**) and DMSA-coated γ-Fe_2_O_3 _nanoparticles (**d**).

The bare and the DMSA-coated γ-Fe_2_O_3 _nanoparticles were both synthesized in our own group (The synthesis process was shown in "Methods" section and the details can be referred to Ref. [5,6]). The nanoparticles were dispersed in pure water, and the pH value was 7. Observed from transmission electron microscopy (TEM) images, the average size of bare nanoparticles was about 11 nm and the DMSA modification seemed to little influence the colloidal size (Figure 1a, b). The hydrodynamic sizes of the bare nanoparticles and the DMSA-coated nanoparticles were about 285 and 103 nm, respectively (Figure 1c, d), meaning that there existed aggregation in both colloidal suspensions more or less. In our experiments, the flux of magnetic field was perpendicular to the substrate supporting colloidal droplet and the field intensity was about 70 kA/m.

**Figure 2:**
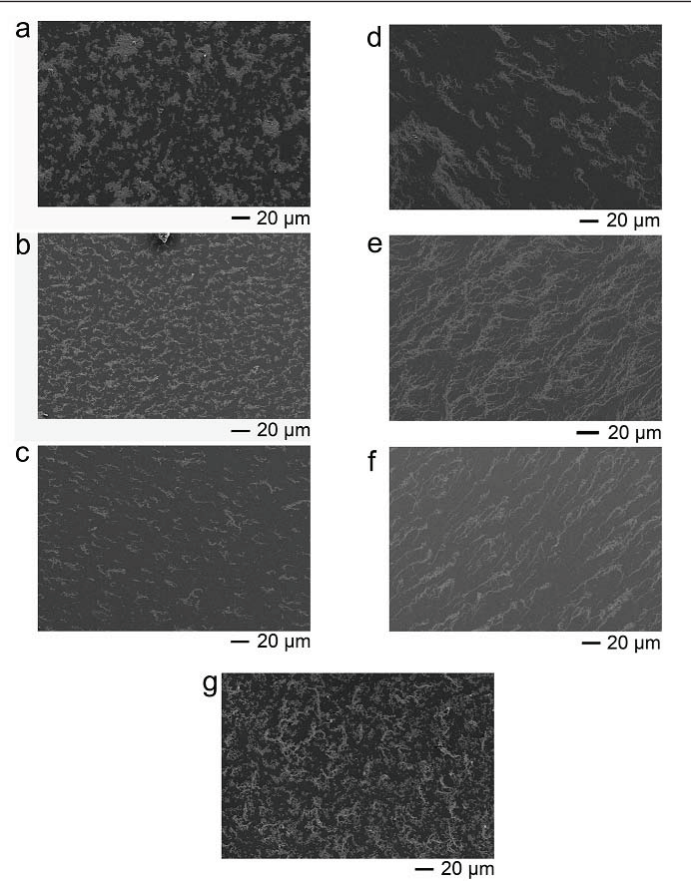
**SEM images of bare γ-Fe_2_O_3 _nanoparticles after solvent drying**. In absence of the alternating magnetic field (**a**) and in presence of alternating magnetic field with different frequency (1 kHz (**b**), 5 kHz (**c**), 10 kHz (**d**), 50 kHz (**e**), 100 kHz (**f**), and 20 Hz (**g**)). The concentration of sample was 12.5 μg/ml. The naturally drying sample showed amorphous aggregates, while the field-treated samples showed more or less one-dimensional orientation. With the frequency increasing, the chain-like assembly was more and more obvious. However, for the 20-Hz alternating magnetic field, the field-treated sample re-showed the amorphous aggregates to some extent, meaning that the alternating magnetic field of the frequency had not induced the assembly of γ-Fe2O3 nanoparticles.

About 4 μL of bare γ-Fe_2_O_3 _colloidal solutions was spread on a silicon wafer and subjected to alternating magnetic field until the solution was dried. In the absence of alternating magnetic field, the solvent drying brought about the amorphous aggregation of γ-Fe_2_O_3 _nanoparticles (Figure 2a). However, when the alternating magnetic field (frequency, 1 K to approximately 100 kHz) was exerted, the nanoparticles formed anisotropic structures (Figure 2b, c, d, e, f). There was a visible transition from amorphous aggregation into fibrous assembly, which reflected the enhancement of magnetic interaction with the frequency increasing. The entropy effect was experimentally excluded to result in the phenomenon because the assembled conformation was found independent upon colloidal concentration (Figure S1 in Additional file 1) [7].

In the presence of magnetic field, the γ-Fe_2_O_3 _nanoparticles will be magnetized and the magnetic moments of nanoparticle can interact with each other. As far as the bare γ-Fe_2_O_3 _nanoparticles are concerned, one cluster of nanoparticles can be magnetized as if it is a large particle. When the external field is time-varied, the magnetic moments of colloidal cluster will also vary with the external field (called magnetic relaxation). Here, the relaxation time of colloidal cluster can be expressed by:(1)

where *τ*_B _is the Brownian relaxation time, *η *is the basic liquid viscosity, *r *is the hydrodynamic radius of the cluster, *k *is the Boltzmann's constant, and *T *is the absolute temperature [5] When the average relaxation time of clusters in colloidal suspension is above the period of external field, the reversal of magnetic moments cannot keep up with the variety of external field, resulting in the occurrence of the anti-parallel magnetic moments to generate the attractive interaction. Based on Equation 1, the relaxation time for 285 nm clusters is 72 ms. Because even the period of 1 kHz field (1 ms) is much below the relaxation time (72 ms), the bare γ-Fe_2_O_3 _nanoparticles can form the one-dimensional assemblies under any kilohertz-ranged alternating magnetic field. Moreover, with the frequency increasing, the magnetic relaxation time of cluster is more and more above the period of external field (The relaxation time is constant while the period of field is the reciprocal of frequency). Then, the magnetic moments of cluster have greater possibility to be perfectly anti-parallel (the angle between two moments is 180°) so that the magnetic interaction between clusters is stronger to overwhelm the disturbances.

According to the abovementioned analysis, when the frequency of external field is low enough, the field will be incapable of inducing the assembly of magnetic nanoparticles. Here, the variety of magnetic moments can keep up with the variety of external field so that the magnetic moments are always parallel, leading to the repulsive interaction. In our experiments, when the frequency of alternating magnetic field was 20 Hz, the visible fibrous assemblies nearly disappeared (Figure 2g). The period of 20-Hz field was 50 ms which has been analogous to the relaxation time. The morphological images of 50 and 100 Hz induced assembly were shown in Additional file 1 (Figure S2). The fibrous assemblies remain able to form. Thus, the assembly mechanism lies in the attractive interaction between anti-parallel magnetic moments, which arises from the incoherent magnetic relaxation of colloidal clusters with respect to the oscillation of field.

**Figure 3:**
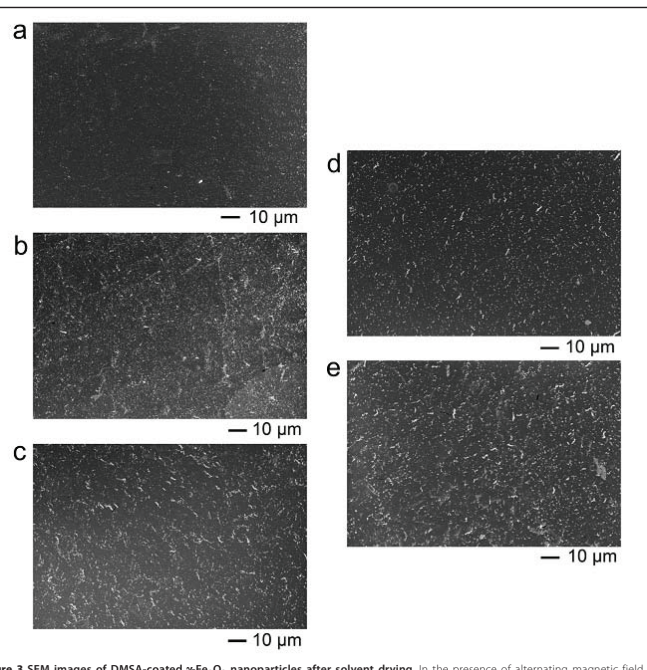
**SEM images of DMSA-coated γ-Fe_2_O_3 _nanoparticles after solvent drying**. In the presence of alternating magnetic field with different frequency (1 kHz (**a**), 5 kHz (**b**), 10 kHz (**c**), 50 kHz (**d**), and 100 kHz (**e**)). The concentration of sample was 12.5 μg/ml. There seemed no obvious difference between samples. In fact, the DMSA-coated nanoparticles cannot be induced to form one-dimensional assemblies by alternating magnetic field with any frequency in our experiments. Thus, the assembly of DMSA-coated nanoparticles seemed little dependent upon the frequency.

**Figure 4:**
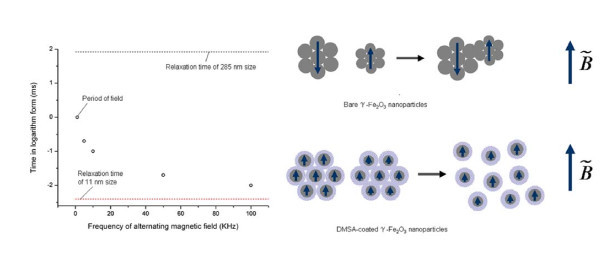
**Schematic illustration of assembly mechanism based on the field periods and the colloidal relaxation time**. If the relaxation time is above the period of field, the assembly can occur. If the relaxation time is below the period of field, there is no attractive force to drive the assembly.

Based on the hydrodynamic size of DMSA-coated γ-Fe_2_O_3 _nanoparticles (Figure 1d), the DMSA-coated nanoparticles should also form the one-dimensional assemblies under the treatment of alternating magnetic field. However, the DMSA-coated nanoparticles actually formed the very small aggregates discretely dispersed on the Si wafer rather than the fibrous assemblies (Figure 3). The magnetic coupling between nanoparticles may account for the phenomenon. Here, the magnetic moments of nanoparticle inside one cluster is unable to merge into a large moment for the DMSA-coated nanoparticles. In the previous work of our group, we found the thickness of DMSA coating layer can be four molecules due to the crosslink of -SH groups [6]. The thick coating layer can hinder the composition of nanoparticulate moments because the dipolar interaction is sharply decreased with the distance between two moments increasing [8]. This hypothesis can be confirmed by comparing the ferromagnetic resonance (FMR) measurement of field-treated sample with that of naturally dried sample (Figure S3 in Additional file 1). For the bare γ-Fe_2_O_3 _nanoparticles, the resonance line width of field-treated sample narrowed evidently with respect to that of naturally dried sample, exhibiting that there exists the magnetic dipolar interaction among the nanoparticles [9]. However, for the DMSA-coated γ-Fe_2_O_3 _nanoparticles, the resonance line width of field-treated sample kept identical, exhibiting that there was no magnetic coupling among the nanoparticles. In this case, the relaxation time should be calculated based on the size of isolated nanoparticle rather than that of nanoparticulate cluster. The relaxation time of 11-nm particle was calculated to be 0.004 ms, far below the periods of external field of any frequency. It means that the variety of magnetic moments of nanoparticle can always keep up with the variety of external field so that the magnetic moments get parallel or approximatively parallel all the while. Since the parallel moments generate repulsive interaction, the final assemblies should be the discrete clusters. Moreover, due to the magnetic repulsive interaction, the size of clusters should be smaller than the original size of aggregates in the suspension. This inference is in accordance with the experimental results. The schematic illustration of assembly mechanism based on the relaxation time with respect to the field period was shown in Figure 4.

## Conclusions

In summary, we demonstrated the frequency response of γ-Fe_2_O_3 _colloidal assembly induced by time-varied magnetic field. The higher frequency favors the formation of fibrous assemblies. The assembly mechanism lies in the difference between the magnetic relaxation time and the field period. It was also preliminarily exhibited that the nanoparticulate assembly induced by alternating magnetic field may be essentially dependent upon the magnetic size rather than the physical size. The work may deepen the knowledge of field-mediated colloidal assembly and widen the technological means for the formation of colloidal patterns.

## Methods

### The synthesis process of bare γ-Fe_2_O_3 _nanoparticles and DMSA-coated γ-Fe_2_O_3 _nanoparticles

#### The synthesis of bare γ-Fe_2_O_3 _nanoparticles

The 25% (*w*/*w*) N(CH_3_)_4_OH was slowly added into the mixture of Fe^2+ ^and Fe^3+ ^(molar ratio is 1:2) until the pH reached 13. Then, the reaction continued for 1 h to obtain the black colloidal particles (Fe_3_O_4_). Then, the air was pumped into the reaction system under the 95°C water bathing after the pH was adjusted to 3. Finally, the reaction system was kept for 3 h to oxidize Fe_3_O_4 _colloidal particles into γ-Fe_2_O_3 _particles. During the whole reaction, the vigorous stirring was needed.

#### The modification of DMSA

The pH and concentration of abovementioned solution were adjusted to 2.7 and 2 mg/ml, respectively. Then, the DMSA molecules were added into the system to react for 5 h. During the whole reaction, the vigorous stirring was needed. Finally, the impurity was removed by dialysis and centrifugation.

## Competing interests

The authors declare that they have no competing interests.

## Authors' contributions

JS and NG initiated the idea. JS carried out the experiments, explained the mechanism, and wrote the manuscript. YS carried out the FMR measurements. CW synthesized both materials. NG constructed the system of time-varied magnetic field.

## Supplementary Material

Additional file 1 **SEM images of bare γ-Fe2O3 nanoparticles after solvent drying**. the assembled conformations of γ-Fe2O3 colloidal solution with different concentrations in the presence of 10 KHz alternating magnetic field. a~d, the concentrations were 12.5 μg/mL, 25 μg/mL, 50 μg/mL and 100 μg/mL, respectively. Figure S2 SEM images of bare γ-Fe2O3 nanoparticles after solvent drying. the assembled conformations of γ-Fe2O3 colloidal solution in the presence of 100 Hz (a) and 50 Hz (b) alternating magnetic field, respectively. The concentration was 12.5 μg/mL. Figure S3 FMR measurements of bare γ-Fe2O3 nanoparticles and DMSA-coatedγ-Fe2O3 nanoparticles with and without field treatment. the ferromagnetic resonance measurements of naturally-dried aggregates and field-treated assemblies. (a), the bare γ-Fe2O3 nanoparticles. (b), the DMSA-coatedγ-Fe2O3 nanoparticles. The resonance line-width denotes the magnetic interaction between nanoparticles. Click here for file
